# Reassessment of the generic limits for *Hydnellum* and *Sarcodon* (Thelephorales, Basidiomycota)

**DOI:** 10.3897/mycokeys.54.35386

**Published:** 2019-06-10

**Authors:** Karl-Henrik Larsson, Sten Svantesson, Diana Miscevic, Urmas Kõljalg, Ellen Larsson

**Affiliations:** 1 Natural History Museum, University of Oslo, P.O. Box 1172 Blindern, NO 0318 Oslo, Norway Gothenburg Global Biodiversity Centre Göteborg Sweden; 2 Gothenburg Global Biodiversity Centre, P.O. Box 461, SE 405 30 Göteborg, Sweden University of Oslo Oslo Norway; 3 Department of Biological and Environmental Sciences, University of Gothenburg, P.O. Box 461, SE 405 30 Göteborg, Sweden University of Gothenburg Göteborg Sweden; 4 Royal Botanic Gardens Victoria, Birdwood Ave, Melbourne, Victoria 3004, Australia Royal Botanic Gardens Victoria Victoria Australia; 5 Västkuststiftelsen, Sandöhamnsvägen 71, SE 434 94 Vallda, Sweden Unaffiliated Vallda Sweden; 6 Institute of Ecology and Earth Sciences, 40 Lai Street, 51005 Tartu, Estonia Institute of Ecology and Earth Sciences Tartu Estonia

**Keywords:** Phylogeny, stipitate hydnoid, taxonomy, *
Thelephorales
*, tooth fungi

## Abstract

DNA sequences from the nuclear LSU and ITS regions were used for phylogenetic analyses of Thelephorales with a focus on the stipitate hydnoid genera *Hydnellum* and *Sarcodon*. Analyses showed that *Hydnellum* and *Sarcodon* are distinct genera but that the current division, based on basidioma texture, makes *Sarcodon* paraphyletic with respect to *Hydnellum*. In order to make genera monophyletic several species are moved from *Sarcodon* to *Hydnellum* and the following new combinations are made: *Hydnellumamygdaliolens*, *H.fennicum*, *H.fuligineoviolaceum*, *H.fuscoindicum*, *H.glaucopus*, *H.joeides*, *H.lepidum*, *H.lundellii*, *H.martioflavum*, *H.scabrosum*, *H.underwoodii*, and *H.versipelle*. Basidiospore size seems to separate the genera in most cases. *Hydnellum* species have basidiospore lengths in the range 4.45−6.95 µm while the corresponding range for *Sarcodon* is 7.4−9 µm. *S.quercinofibulatus* deviates from this pattern with an average spore length around 6 µm. Neotropical *Sarcodon* species represent a separate evolutionary lineage.

## Introduction

**Figure 1.**
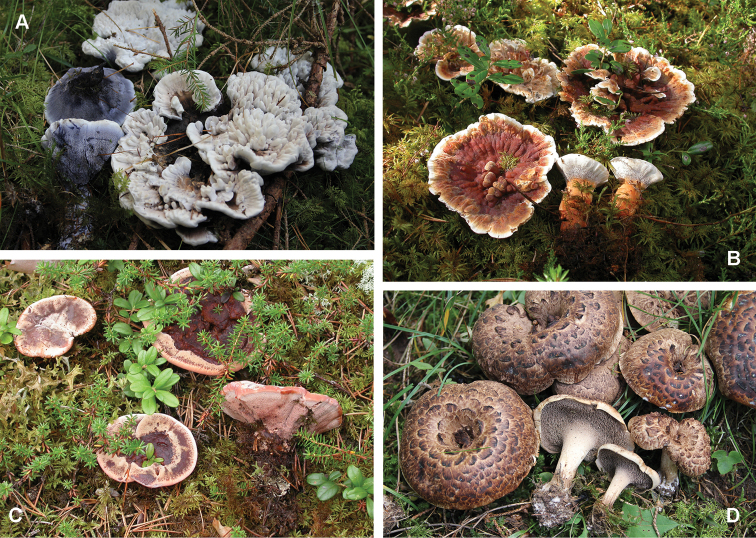
Fruiting bodies of *Hydnellum* and *Sarcodon***A***Hydnellumsuaveolens***B***H.aurantiacum***C***H.ferrugineum***D***Sarcodonimbricatus*.

The order Thelephorales is a distinctive lineage of Agaricomycetes, well-known for its almost ubiquitous ectomycorrhizal life style (Tedersoo et al. 2010). Several species have stipitate hydnoid basidiomata (Fig. 1). They have traditionally been divided into four genera, *Phellodon* and *Bankera* with hyaline basidiospores, and *Hydnellum* and *Sarcodon* with yellow to brown tinted basidiospores (Maas Geesteranus 1975). In both cases the genera within each pair differ in basidioma structure, with *Phellodon* and *Hydnellum* being hard and dry, and *Bankera* and *Sarcodon* forming softer, fleshier basidiomata. This difference in texture is, however, difficult to assess and a series of recent molecular phylogenetic analyses, as outlined below, have indicated that the traditional, morphology-based generic limits are equivocal.

In a recent comprehensive study of stipitate hydnoid species from south-eastern North America, Baird et al. (2013) found that *Bankera* could not be separated from *Phellodon* and the genera were hence combined into a more comprehensive *Phellodon*. The same study suggested that the generic limits of *Sarcodon* and *Hydnellum* need reassessment.

Nitare and Högberg (2012) examined the Nordic species of *Sarcodon* and included a preliminary molecular phylogeny for the species accepted in *Sarcodon*. *Hydnellum* species were also included in non-published test runs and found to be nested among *Sarcodon* species. They concluded that revisions of limits of both genera were probably necessary. Miscevic (2013) expanded on the results in Nitare and Högberg (2012) by including more sequences for each species and by including a selection of *Hydnellum* species in published phylogenies. The results were in congruence with Baird et al. (2013) with regard to overall tree topology and again the conclusion was that the limits of *Sarcodon* and *Hydnellum* need further study. A recent phylogenetic overview of Thelephorales (Vizzini et al. 2016) and a study of *Hydnellum* from the Mediterranean region (Loizides et al. 2016) came to similar conclusions, although Vizzini et al. (2016) did not include sequences from several Neotropical *Sarcodon* species described by Grupe et al. (2015, 2016).

In this paper we analyse ITS and nuclear LSU sequences from a wide selection of Thelephorales species with a focus on *Hydnellum* and *Sarcodon* in order to resolve the relationship between these two genera. We also make some nomenclatural changes that follow from the revision of genus circumscriptions. We demonstrate that Neotropical *Sarcodon* species do not cluster with temperate and boreal species and may be warranted as one or more new genera with more data.

## Methods

For the phylogenetic analyses we compiled two datasets. The first dataset consists of nuclear LSU sequences from most genera in Thelephorales and from a majority of the *Hydnellum* and *Sarcodon* species occurring in Europe. For our two target genera we chose only sequences generated for this study from recently collected basidiomata. We deliberately excluded sequences from specimens identified as *H.concrescens* or *H.scrobiculatum* since these names seem to cover more than just two species and it is currently unclear how the names should be applied (Ainsworth et al. 2010). Since this study is positioned as a revision of the genus limits we were more interested in sequence quality control than a complete coverage of all species reported from Europe.

For our second dataset we chose a different strategy. Here we included ITS sequences from all *Hydnellum* and *Sarcodon* species represented among our own sequences and in GenBank as of December 1, 2018. The reason is that many species, and especially the recently described species from tropical regions, are only available as ITS sequences. However, we made no attempt to verify the identifications given in GenBank and do not endorse them as correct.

DNA was extracted from recent dried collections of basidiomata from North Europe. Voucher numbers, herbarium location, and GenBank numbers are given in Table 1. DNA extraction and PCR protocols follow Larsson et al (2018). Sequencing was either done in-house at University of Oslo, or as a commercial service by Macrogen Inc., South Korea. Assembly of chromatograms was done with Sequencher 5.2.4 (Gene Codes Co., Ann Arbor). Aligning was performed either manually using the editor in PAUP* 4.0a (Swofford 2002) or the software ALIVIEW 1.18 (Larsson 2014), or automatically utilising the L-INS-i strategy as implemented in MAFFT v. 7.017 (Katoh and Standley 2013), followed by manual adjustment.

**Table 1.** Specimens sequenced or downloaded from GenBank. Herbarium acronyms follow Thiers. Sequences generated for this study are marked in bold.

Species	Voucher	Herb.	GenBank number	
			ITS	LSU
*Amaurodonaquicoeruleus* Agerer	Agerer & Bougher	M	AM490944	AM490944
*Amaurodonviridis* (Alb. & Schwein.:Fr.) J.Schröt	KH Larsson 14947b	O	**MK602707**	**MK602707**
*Bankerafuligineoalba* (J.C.Schmidt:Fr.) Pouzar	E Larsson 400-13	GB	**MK602708**	**MK602708**
*Bankeraviolascens* (Alb. & Schwein.:Fr.) Pouzar	MV 130902	GB	**MK602709**	**MK602709**
*Boletopsisleucomelaena* (Pers.:Fr.) Fayod	M Krikorev 140912	GB	**MK602710**	**MK602710**
*Hydnellumaurantiacum* (Batsch:Fr.) P.Karst.	RG Carlsson 08-105	GB	**MK602711**	**MK602711**
* Hydnellum aurantiacum *	E Bendiksen 177-07	O	**MK602712**	**MK602712**
* Hydnellum aurantiacum *	O-F-295029	O	**MK602713**	**MK602713**
*Hydnellumauratile* (Britzelm.) Maas Geest.	O-F-294095	O	**MK602714**	**MK602714**
* Hydnellum auratile *	O-F-242763	O	**MK602715**	**MK602715**
* Hydnellum auratile *	J Nitare 110926	GB	**MK602716**	**MK602716**
*Hydnellumcaeruleum* (Hornem.:Fr.) P.Karst.	O-F-291490	O	**MK602717**	**MK602717**
* Hydnellum caeruleum *	E Bendiksen 575-11	O	**MK602718**	**MK602718**
* Hydnellum caeruleum *	E Bendiksen 584-11	O	**MK602719**	**MK602719**
*Hydnellumcomplicatum* Banker	REB 71		KC571711	
*Hydnellumconcrescens* (Pers.) Banker	K(M)134463	K	EU784267	
*Hydnellumcristatum* (G.F.Atk.) Stalpers	REB 169	TENN	JN135174	
*Hydnellumcumulatum* K.A.Harrison	SE Westmoreland 69		AY569026	
*Hydnellumcyanopodium* K.A.Harrison	SE Westmoreland 85		AY569027	
*Hydnellumdiabolus* Banker	KAH 13873	MICH	AF351863	
*Hydnellumdianthifolium* Loizides, Arnolds & P.-A.Moreau	ML61211HY		KX619419	
*Hydnellumearlianum* Banker	REB 375	TENN	JN135179	
*Hydnellumferrugineum* (Fr.:Fr.) P.Karst.	O-F-297319	O	**MK602720**	**MK602720**
* Hydnellum ferrugineum *	E Larsson 356-16	GB	**MK602721**	**MK602721**
* Hydnellum ferrugineum *	E Larsson 197-14	GB	**MK602722**	**MK602722**
*Hydnellumferrugipes* Coker	REB 176		KC571727	
*Hydnellumgeogenium* (Fr.) Banker	O-F-66379	O	**MK602723**	**MK602723**
* Hydnellum geogenium *	O-F-296213	O	**MK602724**	**MK602724**
* Hydnellum geogenium *	E Bendiksen 526-11	O	**MK602725**	**MK602725**
*Hydnellumgracilipes* (P.Karst.) P.Karst.	E Larsson 219-11	GB	**MK602726**	**MK602726**
* Hydnellum gracilipes *	GB-0113779	GB	**MK602727**	**MK602727**
*Hydnellummirabile* (Fr.) P.Karst.	RG Carlsson 11-119	GB	**MK602728**	**MK602728**
* Hydnellum mirabile *	E Larsson 170-14	GB	**MK602729**	**MK602729**
* Hydnellum mirabile *	S Lund 140912	GB	**MK602730**	**MK602730**
*Hydnellumpeckii* Banker	S Svantesson 328	GB	**MK602731**	**MK602731**
* Hydnellum peckii *	E Larsson 174-14	GB	**MK602732**	**MK602732**
* Hydnellum peckii *	E Bendiksen 567-11	O	**MK602733**	**MK602733**
*Hydnellumpineticola* K.A.Harrison	RB 94		KC571734	
*Hydnellumpiperatum* Maas Geest.	REB 322	TENN	JN135173	
*Hydnellumregium* K.A.Harrison	SE Westmoreland 93		AY569031	
*Hydnellumscleropodium* K.A.Harrison	REB 3	TENN	JN135186	
*Hydnellumscrobiculatum* (Fr.) P.Karst.	REB 78	TENN	JN135181	
*Hydnellumspongiosipes* (Peck) Pouzar	REB 52	TENN	JN135184	
*Hydnellumsuaveolens* (Scop.:Fr.) P.Karst.	E Larsson 139-09	GB	**MK602734**	**MK602734**
* Hydnellum suaveolens *	E Larsson 8-14	GB	**MK602735**	**MK602735**
* Hydnellum suaveolens *	S Svantesson 877	GB	**MK602736**	**MK602736**
*Hydnellumsubsuccosum* K.A.Harrison	REB 10	TENN	JN135178	
*Lenzitopsisdaii* L.W.Zhou & Kõljalg	Yuan 2959	IFP	JN169799	JN169793
*Lenzitopsisoxycedri* Malençon & Bertault	KH Larsson 15304	GB	**MK602774**	**MK602774**
*Odontiafibrosa* (Berk. & M.A.Curtis) Kõljalg	TU115028	TU	**MK602775**	**MK602775**
* Phellodon cf niger *	E Larsson 35-14	GB	**MK602782**	**MK602782**
*Phellodontomentosus* (L.:Fr.) Banker	E Bendiksen 118-10	O	**MK602781**	**MK602781**
*Pseudotomentellaflavovirens* (Höhn. & Litsch.) Svrček	KH Larsson 16190	O	**MK602780**	**MK602780**
*Sarcodonamygdaliolens* Rubio Casas, Rubio Roldán & Català	SC 2011		JN376763	
*Sarcodonaspratus* (Berk.) S.Ito			DQ448877	
*Sarcodonatroviridis* (Morgan) Banker	REB 104	TENN	JN135190	
* Sarcodon atroviridis *	REB 61		KC571768	
*Sarcodonbairdii* A.C.Grupe & Vasco-Pal.	Vasco 990	HUA	KR698938	
*Sarcodoncolombiensis* A.C.Grupe & Vasco-Pal.	Vasco 2084	HUA	KP972654	
*Sarcodonfennicus* (P.Karst.) P.Karst.	S Westerberg 110909	GB	**MK602739**	**MK602739**
* Sarcodon fennicus *	O-F-242833	O	**MK602738**	**MK602738**
* Sarcodon fennicus *	O-F-204087	O	**MK602737**	**MK602737**
*Sarcodonfuligineoviolaceus* (Kalchbr.) Pat.	LA 120818	GB	**MK602740**	**MK602740**
* Sarcodon fuligineoviolaceus *	B Nylén 130918	GB	**MK602741**	**MK602741**
* Sarcodon fuligineoviolaceus *	A Molia 160-2011	O	**MK602742**	**MK602742**
*Sarcodonfuscoindicus* (K.A.Harrison) Maas Geest.	OSC 113622	OSC	EU669228	
*Sarcodonglaucopus* Maas Geest. & Nannf.	RG Carlsson 13-060	GB	**MK602743**	**MK602743**
* Sarcodon glaucopus *	J Nitare 060916	GB	**MK602744**	**MK602744**
* Sarcodon glaucopus *	Å Edvinson 110926	GB	**MK602745**	**MK602745**
*Sarcodonimbricatus* (L.:Fr.) P.Karst.	S Svantesson 355	GB	**MK602748**	**MK602748**
* Sarcodon imbricatus *	J Rova 140829-2	GB	**MK602746**	**MK602746**
* Sarcodon imbricatus *	E Larsson 384-10	GB	**MK602747**	**MK602747**
*Sarcodonjoeides* (Pass.) Bataille	RG Carlsson 11-090	GB	**MK602749**	**MK602749**
* Sarcodon joeides *	K Hjortstam 17589	GB	**MK602750**	**MK602750**
* Sarcodon joeides *	J Nitare 110829	GB	**MK602751**	**MK602751**
* Sarcodon joeides *	REB 270		KC571772	
*Sarcodonlepidus* Maas Geest.	E Grundel 110916	GB	**MK602753**	**MK602753**
* Sarcodon lepidus *	RG Carlsson 10-065	GB	**MK602752**	**MK602752**
* Sarcodon lepidus *	J Nitare 110829	GB	**MK602754**	**MK602754**
*Sarcodonleucopus* (Pers.) Maas Geest. & Nannf.	O-F-296944	O	**MK602756**	**MK602756**
* Sarcodon leucopus *	O-F-296099	O	**MK602755**	**MK602755**
* Sarcodon leucopus *	P Hedberg 080811	GB	**MK602757**	**MK602757**
*Sarcodonlundellii* Maas Geest. & Nannf.	L&A Stridvall 06-049	GB	**MK602758**	**MK602758**
* Sarcodon lundellii *	O-F-242639	O	**MK602759**	**MK602759**
* Sarcodon lundellii *	O-F-295814	O	**MK602760**	**MK602760**
*Sarcodonmartioflavus* (Snell, K.A.Harrison & H.A.C.Jacks.) Maas Geest.	A Delin 110804	GB	**MK602763**	**MK602763**
* Sarcodon martioflavus *	O-F-242435	O	**MK602762**	**MK602762**
* Sarcodon martioflavus *	O-F-242872	O	**MK602761**	**MK602761**
*Sarcodonpakaraimensis* A.C.Grupe & T.W.Henkel	T Henkel 9554	BRG	KM668103	
*Sarcodonpallidogriseus* A.C.Grupe & Vasco-Pal.	Vasco 989	HUA	KR698939	
*Sarcodonportoricensis* A.C.Grupe & T.J.Baroni	TG Baroni 8776	NY	KM668100	
*Sarcodonquercophilus* A.C.Grupe & Lodge	CFMR-BZ-3833	NY	KM668101	
*Sarcodonquercinofibulatus* Pérez-De-Greg., Macau & J.Carbó	JC 20090718-2		JX271818	**MK602773**
*Sarcodonrufobrunneus* A.C.Grupe & Vasco-Pal.	Vasco 1989	HUA	KR698937	
*Sarcodonscabripes* (Peck.) Banker	REB 351	TENN	JN135191	
*Sarcodonscabrosus* (Fr.) P.Karst.	O-F-295824	O	**MK602764**	**MK602764**
* Sarcodon scabrosus *	O-F-292320	O	**MK602766**	**MK602766**
* Sarcodon scabrosus *	O-F-360777	O	**MK602765**	**MK602765**
*Sarcodonsquamosus* (Schaeff.) Quél.	O-F-177452	O	**MK602768**	**MK602768**
* Sarcodon squamosus *	E Larsson 248-12	GB	**MK602767**	**MK602767**
* Sarcodon squamosus *	O-F-295554	O	**MK602769**	**MK602769**
*Sarcodonumbilicatus* A.C.Grupe, T.J.Baroni & Lodge	TJ Baroni 10201	NY	KM668102	
*Sarcodonunderwoodii Banker*	REB 50		KC571781	
*Sarcodonversipellis* (Fr.) Nikol.	RG Carlsson 13-057	GB	**MK602771**	**MK602771**
* Sarcodon versipellis *	RG Carlsson 11-085	GB	**MK602772**	**MK602772**
* Sarcodon versipellis *	E Bendiksen 164-07	O	**MK602770**	**MK602770**
*Sistotremabrinkmannii* (Bres.) J.Erikss.	KH Larsson 14078	GB	KF218967	KF218967
*Steccherinumochraceum* (J.F.Gmel.:Fr.) Gray	KH Larsson 11902	GB	JQ031130	JQ031130
*Thelephoracaryophyllea* (Schaeff.:Fr.) Pers.	E Larsson 89-09S	GB	**MK602776**	**MK602776**
*Thelephoraterrestris* Ehrh.:Fr.	E Larsson 295-13	GB	**MK602777**	**MK602777**
*Tomentellastuposa* (Link) Stalpers	Th-0764	O	**MK602778**	**MK602778**
*Tomentellopsispulchella* Kõljalg & Bernicchia	KH Larsson 16366	O	**MK602779**	**MK602779**

In the phylogenetic analyses we assumed the following minimal partitions for the nrDNA region: ITS1, 5.8S, ITS2 and LSU (approximately 1200 bases of the 5’ end). Two datasets were analysed separately: an LSU dataset only including the LSU region, and an ITS dataset including ITS1, 5.8S and ITS2. We used the automated best-fit tests implemented in PAUP* 4.0a (Swofford 2002) to select optimal substitution models for each complete, non-partitioned dataset (PHYML) and optimal substitution model partitions for each minimal partition (BEAST). Models and partitions were chosen based on BIC score for the BEAST analysis and AICc score for the PHYML analysis. All tests were conducted using three substitution schemes and evaluated substitution models with equal and gamma-distributed among-site rate variation. The tests for the PHYML analysis also evaluated substitution models with invariant sites. The following partitions and models had the highest ranking, according to BIC: ITS1+ITS2 (GTR+G), 5.8S (K80+G), LSU (GTR+G). According to AICc the GTR+I+G model provided the best fit for both the ITS and the LSU datasets.

To generate Bayesian phylogenetic trees (BI) from the alignments we used BEAST 2.4.7 (Bouckaert et al. 2014). We prepared the xml-files for the BEAST 2 runs in BEAUTI 2.4.7 (Bouckaert et al. 2014). We set the substitution model to GTR+G for the LSU run. In the ITS run we set it to HKY+G for 5.8S, since it is the most similar model to K80+G available in the program. Test runs revealed convergence problems due to insufficient data for some substitution rates in the GTR+G model initially used for the ITS1+ITS2 partition, and it was hence changed to HKY+G. In the ITS run the substitution rate of both partitions were estimated independently. We set the trees of the minimal nrDNA partitions as linked in this analysis and the clock models as unlinked. A lognormal, relaxed clock model was assumed for each partition, as test runs had shown that all partitions had a coefficient of variation well above 0.1 (i.e. implying a relatively high rate variation among branches). The clock rate of each partition was estimated in the runs, using a lognormal prior with a mean set to one in real space. We set the growth rate prior to lognormal, with a mean of 5 and a standard deviation of 2. We ran the Markov Chain Monte Carlo (MCMC) chains of both datasets for 20 million generations with tree and parameter files sampled every 1,000 generations. The analyses all converged well in advance of the 10 % burn-in threshold, had ESS values well above 200 for all parameters, and chain mixing was found to be satisfactory as assessed in TRACER 1.6.0 (Rambaut et al. 2014). After discarding the burn-in trees, maximum clade credibility trees were identified by TREEANNOTATOR 2.4.7 (Bouckaert et al. 2014).

To generate Maximum Likelihood (ML) gene trees we used PHYML 3.1 (Guindon et al. 2010). We set the substitution model to GTR+I+G for both the ITS and LSU datasets. Tree topology search was conducted using NNI+SPR, with ten random starting trees. Non-parametric bootstrap analyses with 1000 replicates were performed on the resulting trees.

## Results

Seventy-five Thelephorales specimens from the genera *Amaurodon*, *Bankera*, *Boletopsis*, *Hydnellum*, *Lenzitopsis*, *Phellodon*, *Pseudotomentella*, *Sarcodon*, *Thelephora*, *Tomentella*, and *Tomentellopsis*, were sequenced for this study. In addition, 39 sequences were downloaded from public databases (GenBank, UNITE) including outgroup sequences of *Steccherinumochraceum* (Polyporales) and *Sistotremabrinkmannii* (Cantharellales) included in the LSU dataset. The ITS analyses were rooted by the default method (BEAST) or left unrooted (PHYML).

The aligned LSU dataset consisted of 1443 nucleotide positions. After exclusion of ambiguous regions 1377 positions remained for the analyses. BI returned a tree where the focus genera *Hydnellum* and *Sarcodon* are distributed over two strongly supported clades. The larger of these clades includes the type of *Hydnellum*, *H.suaveolens*, and an additional 17 species, all except one forming strongly supported terminal clades. Nine of these taxa are currently placed in *Sarcodon*. With a few exceptions the relationships within *Hydnellum* are not resolved. *H.aurantiacum* and *H.auratile* are recovered as a strongly supported group; *Sarcodonscabrosus* and *S.fennicus* are grouped with 0.97 posterior probability support; *S.fuligineoviolaceus*, *S.glaucopus*, and *S.joeides* form a subclade with 0.97 posterior probability support; and finally *H.suaveolens* and *S.versipellis* form a strongly supported clade. The type of *Sarcodon*, *S.imbricatus*, and three other species form the second main clade. The three sequences of *S.imbricatus* cluster together but the clade is unsupported. *Hydnellum* and *Sarcodon* are recovered as sister clades but the support for this arrangement is weak.

**Figure 2.**
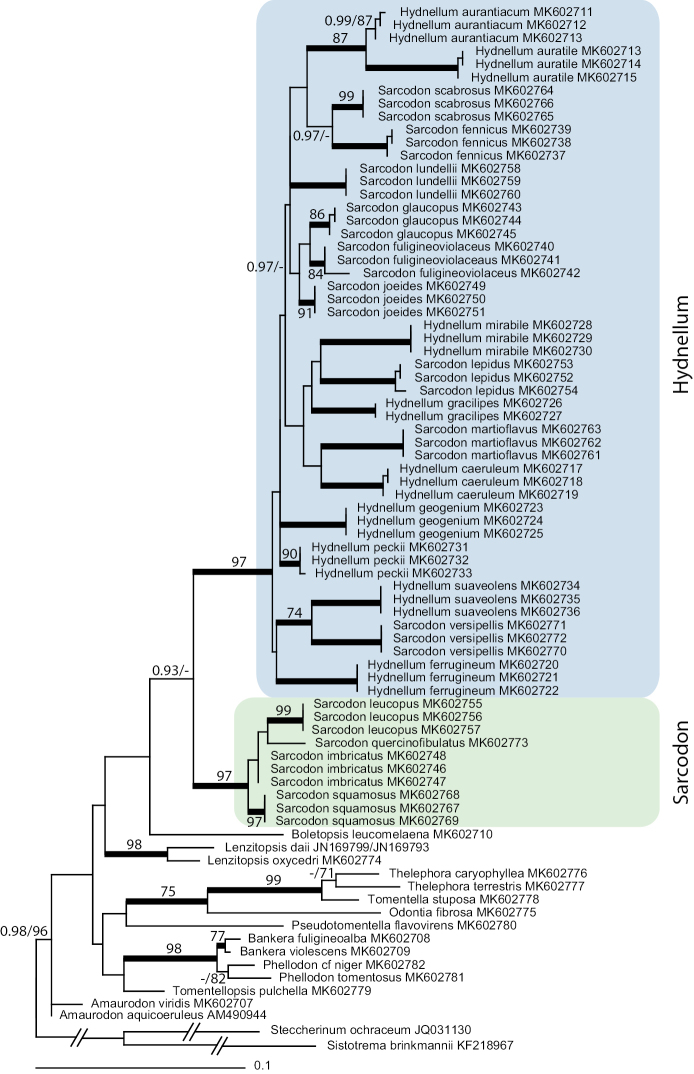
Maximum likelihood analyses of LSU dataset for Thelephorales. Branches in bold have a posterior probability value of 1 in Bayesian inference and 100% bootstrap support in ML analysis, if not otherwise indicated by a figure. Lower support values on other branches are indicated by figures. *Steccherinumochraceum* and *Sistotremabrinkmannii* are used as outgroup (branch lengths shortened).

For target taxa the ML tree is essentially similar to the BI tree with strong support for the similarly composed *Hydnellum* and *Sarcodon* clades (Fig. 2). As for the BI analysis the relationships among species within *Hydnellum* and *Sarcodon* are not resolved except for a weak to moderate support for grouping *H.aurantiacum* with *H.auratile* and *H.suaveolens* with *S.versipellis*. *S.fuligineoviolaceus*, *S.glaucopus*, and *S.joeides* also group together in the ML tree but without support. Again *S.imbricatus* does not get support and is not separated from *S.quercinofibulatus*.

**Figure 3.**
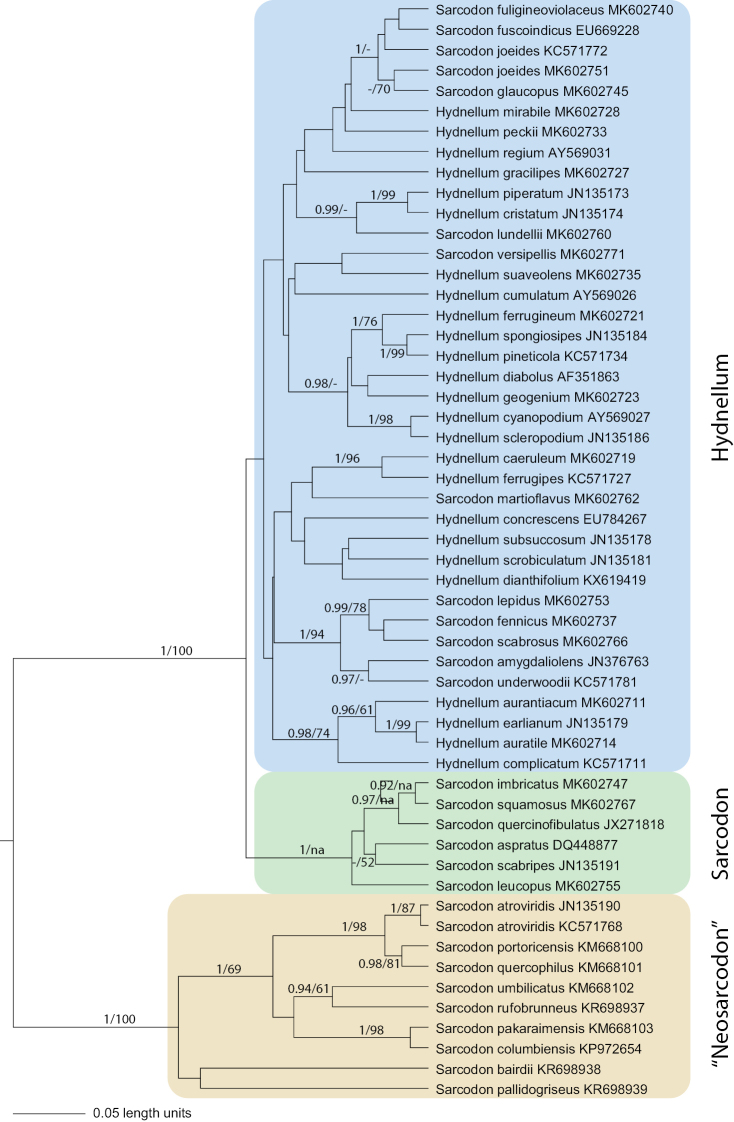
Ultrametric default rooted BEAST tree of ITS dataset for *Hydnellum* and *Sarcodon*. Posterior probability values and bootstrap percent support from ML analysis are indicated by figures; na = not applicable.

The aligned ITS dataset consisted of 1068 nucleotide positions of which 505 remained for the analyses after removal of ambiguous regions. Bayesian inference produced a tree with two strongly supported clades (Fig. 3). The smaller one, which we here informally call “Neosarcodon”, contains nine *Sarcodon* species, all with a distribution in the tropical and subtropical Americas. Remaining *Hydnellum* and *Sarcodon* taxa, including both type species, formed the other clade. Within the latter clade two subclades are visible, corresponding to the genera *Hydnellum* and *Sarcodon*, and with the same delimitation as in the LSU trees. Only the *Sarcodon* subclade has strong support. Within each larger clade several groups of taxa received moderate to strong support. The reader is referred to Fig. 2 for further details.

The ML tree recovered the same two main clades with strong support but could not resolve the relationships within the larger *Hydnellum*/*Sarcodon* clade. In the ML tree the clade corresponding to *Hydnellum* in the LSU tree is correctly identified but not supported while the clade corresponding to *Sarcodon* appears polyphyletic.

Based on these results we hereby revise the limits of the two genera by moving a number of species from *Sarcodon* to *Hydnellum*. Consequently the genus description for *Hydnellum* must be emended while the genus description for *Sarcodon* can remain unaltered.

### Taxonomy

#### Hydnellum

Taxon classificationFungiThelephoralesBankeraceae

P.Karst., Meddn Soc. Fauna Flora fenn. 5: 41 (1879).

##### Type species.

*Hydnellumsuaveolens* (Scop.:Fr.) P.Karst. (1879)

##### Basionym.

*Hydnumsuaveolens* Scop.:Fr. (1772)

Basidiomata with pileus and stipe, single or concrescent; pileus thin to thick, at first smooth and velutinous, when mature felted, fibrillose, scaly, ridged, or irregularly pitted and scrupose, mostly brownish but also with white, olive yellowish, orange, purplish or bluish colours, often concentrically zonate; stipe narrow to thick, solid, mostly short; hymenophore hydnoid, usually strongly decurrent; context from soft and brittle to corky or woody; hyphal system monomitic, septa with or without clamps, context hyphae inflated or not; cystidia lacking; basidia narrowly clavate, producing four sterigmata; basidiospores with irregular outline, more or less lobed, verrucose, brownish. Terrestrial, forming ectomycorrhiza with forest trees.

#### Hydnellum amygdaliolens

Taxon classificationFungiThelephoralesBankeraceae

(Rubio Casas, Rubio Roldán & Català) E.Larss., K.H.Larss. & Kõljalg comb. nov.

830570

##### Basionym.

*Sarcodonamygdaliolens* Rubio Casas, Rubio Roldán & Català, Boln Soc. Micol. Madrid 35: 44−45. 2011. Holotype: Spain, Tamajón, Barranco la Jara. L. Rubio-Casas & L. Rubio-Roldán, AH 42113.

#### Hydnellum fennicum

Taxon classificationFungiThelephoralesBankeraceae

(P.Karst.) E.Larss., K.H.Larss. & Kõljalg comb. nov.

830571

##### Basionym.

Sarcodonscabrosusvar.fennicus P.Karst., Bidr. Känn. Finl. Nat. Folk 37: 104. 1882. Type: not indicated (neotype: H, designated by Maas Geesteranus & Nannfeldt 1969: 406)

#### Hydnellum fuligineoviolaceum

Taxon classificationFungiThelephoralesBankeraceae

(Kalchbr.) E.Larss., K.H.Larss. & Kõljalg comb. nov.

830572

##### Basionym.

*Hydnumfuligineoviolaceum* Kalchbr., in Fries, Hymenomyc. eur. (Upsaliae): 602. 1874. Holotype: Slovakia, Presovsky kraj, Olaszi. C. Kalchbrenner, UPS F-173546.

#### Hydnellum fuscoindicum

Taxon classificationFungiThelephoralesBankeraceae

(K.A.Harrison) E.Larss., K.H.Larss. & Kõljalg comb. nov.

830573

##### Basionym.

*Hydnumfuscoindicum* K.A.Harrison, Can. J. Bot. 42: 1213. 1964. Holotype: USA, Washington, Olympic Nat. Park, A.H. Smith. MICH 10847.

#### Hydnellum glaucopus

Taxon classificationFungiThelephoralesBankeraceae

(Maas Geest. & Nannf.) E.Larss., K.H.Larss. & Kõljalg comb. nov.

830574

##### Basionym.

*Sarcodonglaucopus* Maas Geest. & Nannf., Svensk bot. Tidskr. 63: 407. 1969. Holotype: Sweden, Uppland, Börje par., J. Eriksson. UPS F-013955.

#### Hydnellum joeides

Taxon classificationFungiThelephoralesBankeraceae

(Pass.) E.Larss., K.H.Larss. & Kõljalg comb. nov.

830575

##### Basionym.

*Hydnumjoeides* Pass., Nuovo G. bot. ital. 4: 157. 1872. Holotype: Italy, Emilia-Romagna, Collecchio, G. Passerini. PAD.

#### Hydnellum lepidum

Taxon classificationFungiThelephoralesBankeraceae

(Maas Geest.) E. Larss., K.H.Larss. & Kõljalg comb. nov.

830576

##### Basionym.

*Sarcodonlepidus* Maas Geest., Verh. K. ned. Akad. Wet., tweede sect. 65: 105. 1975. Holotype: The Netherlands, Lochem, Ampsen, G. & H. Piepenbroek. L.

#### Hydnellum lundellii

Taxon classificationFungiThelephoralesBankeraceae

(Maas Geest. & Nannf.) E.Larss., K.H.Larss. & Kõljalg comb. nov.

830577

##### Basionym.

*Sarcodonlundellii* Maas Geest. & Nannf., Svensk bot. Tidskr. 63: 421. 1969. Type: Sweden, Uppland, Storvreta, S. Lundell & J.A. Nannfeldt, distributed in S. Lundell & J.A. Nannfeldt Fungi exs. suec. as number 252 (lectotype, designated here, UPS F-010975; MycoBank No.: MBT387081). The UPS herbarium has two copies of the exsiccate and the specimens of *H.lundellii* are registered as F-010975 and F-013956, respectively. From F-010975 an ITS2 sequence has been generated [GenBank MK753037] and this specimen is here selected as lectotype).

#### Hydnellum martioflavum

Taxon classificationFungiThelephoralesBankeraceae

(Snell, K.A.Harrison & H.A.C.Jacks.) E.Larss., K.H.Larss. & Kõljalg comb. nov.

830578

##### Basionym.

*Hydnummartioflavum* Snell, K.A.Harrison & H.A.C.Jacks., Lloydia 25: 161. 1962. Holotype: Canada, Quebec, Ste Anne de la Pocatière, H.A.C. Jackson & W.H. Snell 13 Sep. 1954, BPI 259438.

#### Hydnellum scabrosum

Taxon classificationFungiThelephoralesBankeraceae

(Fr.) E.Larss., K.H.Larss. & Kõljalg comb. nov.

830579

##### Basionym.

*Hydnumscabrosum* Fr., Anteckn. Sver. Ätl. Svamp.: 62. 1836. Type: not indicated (neotype: Sweden, Småland, Femsjö, S. Lundell, UPS F-013954, designated by Maas Geesteranus & Nannfeldt 1969: 426)

#### Hydnellum underwoodii

Taxon classificationFungiThelephoralesBankeraceae

(Banker) E.Larss., K.H.Larss. & Kõljalg comb. nov.

830580

##### Basionym.

*Sarcodonunderwoodii* Banker, Mem. Torrey bot. Club 12: 147. 1906. Holotype: USA, Connecticut, NY 776131.

#### Hydnellum versipelle

Taxon classificationFungiThelephoralesBankeraceae

(Fr.) E.Larss., K.H.Larss. & Kõljalg comb. nov.

830581

##### Basionym.

*Hydnumversipelle* Fr., Öfvers. K. Svensk. Vetensk.-Akad. Förhandl. 18(1): 31. 1861. Type: not indicated (neotype: Sweden, Uppland, Danmark par., J. Eriksson & H. Nilsson, UPS F-013958, designated by Maas Geesteranus & Nannfeldt 1969: 430)

#### Sarcodon

Taxon classificationFungiThelephoralesBankeraceae

Quél. ex P.Karst., Revue mycol., Toulouse 3 (no. 9): 20 (1881).

##### Type species.

*Sarcodonimbricatus* (L.:Fr.) P.Karst. (1881)

##### Basionym.

*Hydnumimbricatum* L.:Fr. (1753).

Basidiomata with pileus and stipe, single or concrescent; pileus thin to thick, at first smooth and velutinous, when mature smooth or scaly, brownish; stipe thick, solid, mostly short; hymenophore hydnoid, usually strongly decurrent; context soft and brittle; hyphal system monomitic, septa with clamps, context hyphae inflated; cystidia lacking; basidia narrowly clavate, producing four sterigmata; basidiospores with irregular outline, more or less lobed, verrucose, brownish. Terrestrial, forming ectomycorrhiza with forest trees.

## Discussion

In this paper we show that the current morphology-based concepts of *Sarcodon* and *Hydnellum* do not correspond to monophyletic subgroups within the Thelephorales. The characters traditionally used to separate the two genera do not reflect true relationships. These characters, however, are vague and open to subjectivity; hence it is not surprising that they have now been shown to be unreliable. Maas Geesteranus (1975) pointed to the context structure and consistency as the main differentiating character. For *Hydnellum* he describes the context as “... fibrillose, soft or tough, corky to woody, more or less duplex, zoned, ...” and hyphae are said to be “...usually not inflating ...”. In *Sarcodon* the same structures are described as “... fleshy, brittle, soft or firm (never corky or woody), not duplex, not zoned ...” and “...hyphae inflating ...”. While these morphological characteristics remain true for *Sarcodon*, the corresponding descriptions for *Hydnellum* had to be emended.

Instead of context structure it seems that average basidiospore size may in most cases offer a possibility to separate a *Sarcodon* species from one belonging to *Hydnellum*. Table 2 summarizes basidiospore measurements from the literature. Average basidiospore lengths in *Hydnellum* fall between 4.45 and 6.95 µm while the same figures for *Sarcodon* are 7.4 and 9 µm, ornamentation excluded. However, *S.quercinofibulatus* clearly deviates from this pattern. According to measurements in the protologue (Pérez-de-Gregorio et al. 2011) and in Vizzini et al. (2013) average basidiospore length was measured to 6.95 and 7.0, respectively, but then included the ornamentation. Measurements excluding ornamentation would be approximately 1 µm less. Clearly, for *S.quercinofibulatus* basidiospore length alone will not be decisive for genus placement.

**Table 2.** Basidiospore measurements for *Hydnellum* and *Sarcodon* from the literature. Sources: B = Baird et al. (2013), M = Maas Geesteranus (1975), J = Johannesson et al. (1999). All measurements exclude ornamentation. For species treated in this paper names follow our new classification. For other species names are according to cited authors.

**Species**	**Measurements**	**Mean length**
*Hydnellumaurantiacum* (M)	(5.8−)6−6.7 × (4−)4.3−4.9	6.35
*Hydnellumauratile* (M)	4.9−5.8 × 3.6−4.5	5.35
*Hydnellumcaeruleum* (M)	5.4−6(−6.3) × 3.4−4.3	5.70
*Hydnellumcompactum* (Pers.:Fr.) P.Karst. (M)	5.4−6.3 × 3.6−4.5	5.85
*Hydnellumcomplicatum* (B)	4−5 × 3−5	4.50
*Hydnellumconcrescens* (M)	5.4−6.1 × (3.6−)4−4.5	5.75
*Hydnellumcristatum* (B)	5−6 × 4−5	5.50
*Hydnellumcruentum* K.A.Harrison (B)	4−5 × 3−4	4.50
*Hydnellumcumulatum* (M)	4.3−5.6 × 3.6−4.3	4,95
*Hydnellumdiabolus* (B)	6−7 × 5−6	6.50
*Hydnellumearlianum* (B)	5−6 × 4−5	5.50
*Hydnellumfennicum* (M)	6.3−7.6 × 4.5−5.2	6.95
*Hydnellumferrugineum* (M)	(5.4−)5.8−6.3 × 3.6−4.5	6.05
*Hydnellumferrugipes* (B)	5−7 × 5−6	6.00
*Hydnellumfuligineoviolaceum* (M)	5.4−6.5 × 4−4.7(−5.4)	5.95
*Hydnellumgeogenium* (M)	4.5−5.2 × 3.1−3.6	4.85
*Hydnellumglaucopus* (M)	(5−)5.4−5.8(−6.3) × (3.6−)4−4.5	5.60
*Hydnellumgracilipes* (M)	4.3−4.6 × 2.7−3.6	4.45
*Hydnellumjoeides* (M)	5.4−5.8 × 3.6−4.2	5.60
*Hydnellumlepidum* (M)	5.8−6.3 × 3.6−4.3	6.05
*Hydnellumlundellii* (M)	4.9−5.8 × 3.6−4.2	5.35
*Hydnellummartioflavum* (M)	5−6.3 × 3.6−4.5	5.65
*Hydnellumpeckii* (M)	4.9−5.4 × 3.8−4	5.15
*Hydnellumpineticola* (B)	5−7 × 4−6	6.00
*Hydnellumpiperatum* (B)	4−6 × 4−5	5.00
*Hydnellumscabrosum* (M)	(5.4−)6.3−7.3 × (3.6−)4−5	6.80
*Hydnellumscleropodium* (B)	4−6 × 3−4	5.00
*Hydnellumspongiosipes* (B)	6−7 × 5−6	6.50
*Hydnellumsuaveolens* (M)	4−5 × 3−3.6	4.50
*Hydnellumsubsuccosum* (B)	5−6 × 4−6	5.50
*Hydnellumversipelle* (M)	4.5−5.5 × 3.5−4.5	5.00
*Hydnellumunderwoodii* (B)	5−7 × 5−6	6.00
*Sarcodonatroviridis* (B)	8−9 × 7−8	8.50
*Sarcodonexcentricus* R.E.Baird (B)	8−9 × 6−8	8.50
*Sarcodonharrisonii* R.E.Baird (B)	7−9 × 6−8	8.00
*Sarcodonleucopus* (M)	(6.7−)7.2−7.6(−9) × 4.5−5.6	7.40
*Sarcodonimbricatus* (M)	7.2−8.2 × 4.9−5.4	7.70
*Sarcodonscabripes* (B)	8−10 × 7−9	9.00
*Sarcodonsquamosus* (J)	7.2−8.2 × 4.9−5.4	7.70

Not all sequences from species described as *Sarcodon* spp. were recovered within either *Sarcodon* or *Hydnellum*. In our ITS-only analyses nine species formed a well-supported clade of their own, separated from *Sarcodon* sensu stricto and *Hydnellum* (Fig. 3). This clade, here informally called “Neosarcodon”, contains species collected in tropical and subtropical regions of the Western Hemisphere and may represent one or several distinct genera. However, further analyses based on an expanded dataset using more conservative molecular markers would be required to definitely identify any new higher taxa in the group.

The failure to generate support for *Sarcodon* and *Hydnellum* in the ITS-only analyses reflects the large genetical distances present among the species within this marker. Our general experience with the ITS region for thelephoralean target genera is that species are extremely well separated and the internal variation surprisingly low, even when a large number of specimens from both Europe and America are considered. On the other hand, the genetical difference among species is moderate to high, making alignments difficult and prone to ambiguities. In our ITS analyses we chose to remove ambiguous regions, thus halving the number of nucleotide positions suggested by automatic alignment through MAFFT. This seems to have affected the ML analyses most. However, the ITS analyses only served to position neotropical *Sarcodon* species and the results clearly show that they belong to a separate lineage.

Otto (1997) suggested that *Hydnumauratile* is a later synonym of *Hydnumaurantiacum* and that the species we now call *Hydnellumaurantiacum* should be named *Hydnellumfloriforme* (Schaeff.) Banker. The name change is based on a reinterpretation of Batsch’s original illustration, which, according to Otto, clearly shows the same species as *Hydnumauratile*. In phylogenetic analyses *H.aurantiacum* and *H.auratile* are sister taxa and during our study we have sequenced several specimens identified as *H.auratile* that turned out to be *H.aurantiacum*. Thus separating these species can be hazardous and to interpret illustrations must be even harder. We currently do not accept this unfortunate name change.

The present study will serve as the basis for further exploration of species limits within *Hydnellum* and *Sarcodon*. As has been demonstrated for the genera, many species interpretations are in need of revision. Over the years we have found numerous specimen misidentifications as well as specimens that could not be assigned to pre-existing names. A closer inspection of the ITS tree in Fig. 3, where we let the terminals retain the identifications given in GenBank, shows some examples. The American sequence of *Sarcodonjoeides* (KC571772) does not cluster with the European representative of the same species (MK602751) and the American sequence named *Hydnellumearlianum* seems to be identical to what is in Europe called *H.auratile*. Considering that many stipitate hydnoid species are red-listed and used as indicators of forests in need of conservation (Ainsworth 2005, Nitare 2019), it is of utmost importance to sort out the taxonomy of these species.

## Supplementary Material

XML Treatment for Hydnellum

XML Treatment for Hydnellum amygdaliolens

XML Treatment for Hydnellum fennicum

XML Treatment for Hydnellum fuligineoviolaceum

XML Treatment for Hydnellum fuscoindicum

XML Treatment for Hydnellum glaucopus

XML Treatment for Hydnellum joeides

XML Treatment for Hydnellum lepidum

XML Treatment for Hydnellum lundellii

XML Treatment for Hydnellum martioflavum

XML Treatment for Hydnellum scabrosum

XML Treatment for Hydnellum underwoodii

XML Treatment for Hydnellum versipelle

XML Treatment for Sarcodon
